# Oligomerization domains in the glycan‐binding receptors DC‐SIGN and DC‐SIGNR: Sequence variation and stability differences

**DOI:** 10.1002/pro.3083

**Published:** 2016-12-22

**Authors:** Ália dos Santos, Andreas Hadjivasiliou, Felipe Ossa, Novandy K. Lim, Aylin Turgut, Maureen E. Taylor, Kurt Drickamer

**Affiliations:** ^1^ Department of Life SciencesImperial CollegeLondonSW7 2AZUnited Kingdom

**Keywords:** glycan‐binding receptor, C‐type lectin, multivalency, oligomerization domains, coiled coil

## Abstract

Human dendritic cell‐specific intercellular adhesion molecule‐1 grabbing nonintegrin, DC‐SIGN, and the sinusoidal endothelial cell receptor DC‐SIGNR or L‐SIGN, are closely related sugar‐binding receptors. DC‐SIGN acts both as a pathogen‐binding endocytic receptor and as a cell adhesion molecule, while DC‐SIGNR has only the pathogen‐binding function. In addition to differences in the sugar‐binding properties of the carbohydrate‐recognition domains in the two receptors, there are sequence differences in the adjacent neck domains, which are coiled‐coil tetramerization domains comprised largely of 23‐amino acid repeat units. A series of model polypeptides consisting of uniform repeat units have been characterized by gel filtration, differential scanning calorimetry and circular dichroism. The results demonstrate that two features characterize repeat units which form more stable tetramers: a leucine reside in the first position of the heptad pattern of hydrophobic residues that pack on the inside of the coiled coil and an arginine residue on the surface of the coiled coil that forms a salt bridge with a glutamic acid residue in the same polypeptide chain. In DC‐SIGNR from all primates, very stable repeat units predominate, so the carbohydrate‐recognition domains must be held relatively closely together. In contrast, stable repeat units are found only near the membrane in DC‐SIGN. The presence of residues that disrupt tetramer formation in repeat units near the carbohydrate‐recognition domains of DC‐SIGN would allow these domains to splay further apart. Thus, the neck domains of DC‐SIGN and DC‐SIGNR can contribute to the different functions of these receptors by presenting the sugar‐binding sites in different contexts.

## Summary for Broader Audience

The spacing between sugar‐binding sites in receptors of the innate immune system contributes to the way that these receptors distinguish sugars on the surface of pathogens from those on host cells. This work describes how subtle differences in closely related receptors can change the way that sugar‐binding sites are held together, helping to explain why they interact differently with viruses that display similar types of sugars.

## Introduction

Human DC‐SIGN, the dendritic cell‐specific intercellular adhesion molecule‐1 grabbing nonintegrin, plays a dual role as an adhesion receptor for T cells and as a pathogen‐binding receptor.^1^, ^2^ The cell adhesion function is probably associated with the ability of the receptor to bind the Lewis^x^ trisaccharide found on cell surface molecules, including ICAM‐1. Pathogens bound by the receptor include viruses and fungi, which bear high mannose oligosaccharides, as well as parasites that are rich in the Lewis^x^ epitope. The dual binding specificity can be explained at least in part by accommodation of both Lewis^x^ and high mannose oligosaccharides in the binding site of the C‐type carbohydrate recognition domain (CRD) that forms the ligand‐binding portion of the receptor.^3^ In contrast, the closely related human receptor DC‐SIGNR shows more restricted binding to the high mannose oligosaccharides, allowing it to interact with viral pathogens.^4^ This receptor is also designated L‐SIGN, reflecting its expression on sinusoidal endothelial cells of lymph nodes and liver, as well as on specific cell types in the placenta.

**Figure 1:**
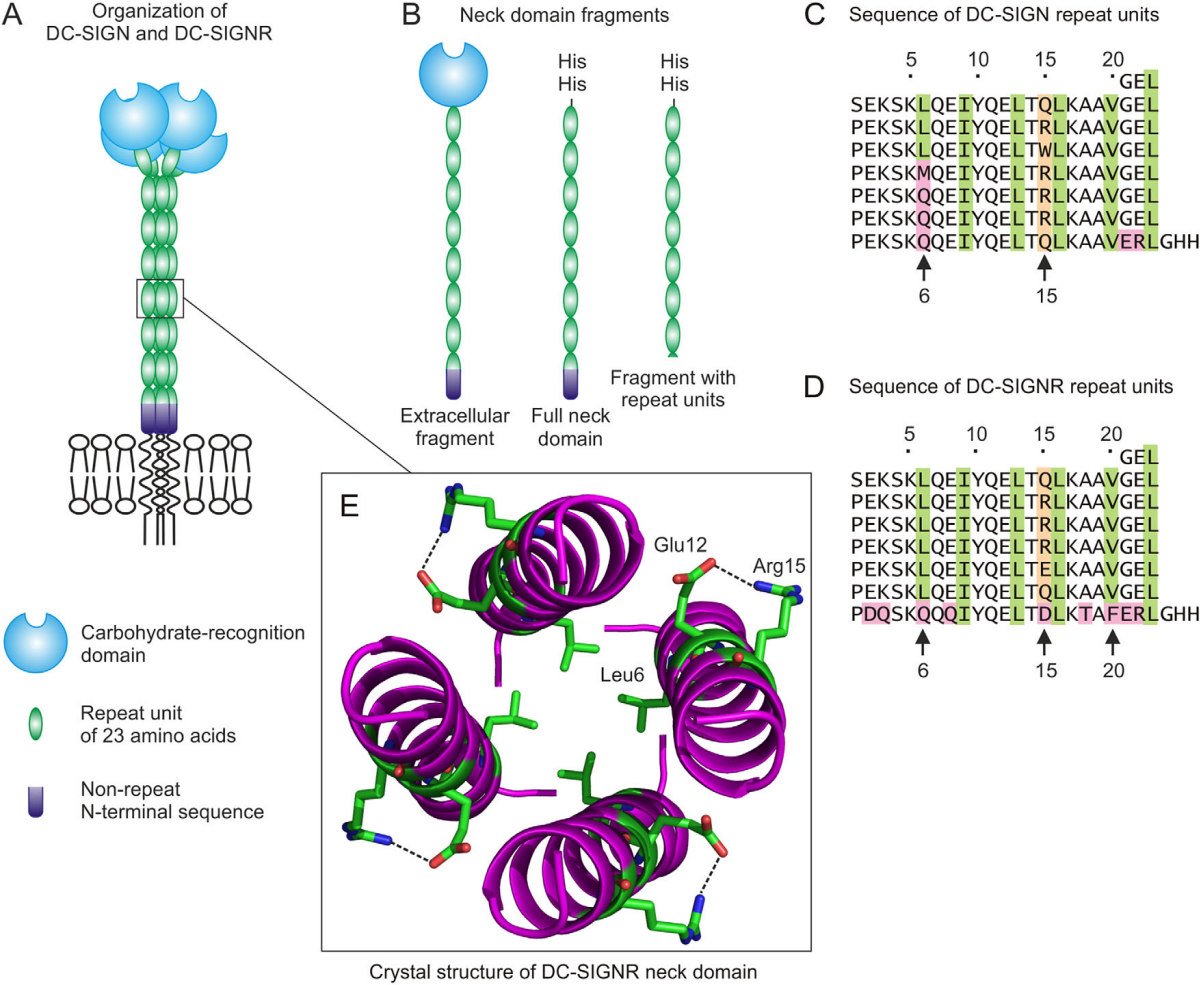
(A) Domains in the extracellular portions of human DC‐SIGN and DC‐SIGNR. (B) The histidine‐tagged full length neck fragment of DC‐SIGN is shown, along with a truncated fragment consisting of seven repeat units of 23‐amino acids. This truncated neck domain corresponds to the portion of DC‐SIGNR that was previously crystallized.^5^ (C,D) Sequences of the repeat units from the neck domains of human DC‐SIGN and DC‐SIGNR. The hydrophobic amino acids that form a heptad pattern in each repeat unit are highlighted in green, variable residues at position 15 are highlighted in peach and other positions that diverge from the sequence pattern are highlighted in pink. (E) Structure of the neck domain of DC‐SIGNR, showing the position of the leucine residue at position 6 inside the coiled‐coil structure and the hydrogen bond between the glutamic acid residue at position 12 and the arginine residue at position 15 on the surface of the helix. Figure is based on Protein Data Bank entry 3JQH and was generated with PyMOL.

DC‐SIGN and DC‐SIGNR are similar in overall organization, consisting of tetramers that facilitate binding to multivalent targets, such as viral and cell surfaces. In both proteins, clusters of C‐terminal CRDs are projected from the cell surface by extended neck domains that consists largely of multiple 23‐amino acid repeat units (Fig. 1). Information about the mechanisms by which the CRDs in DC‐SIGN and DC‐SIGNR are held together in oligomers is of interest because differences in the geometry of binding site clustering is believed to underlie differences in the ability of human immunodeficiency virus (HIV), West Nile virus, and other viruses to bind and infect cells expressing these receptors.^6^, ^7^, ^8^ Recent studies with mannose‐capped quantum dots have provided further evidence that the arrangement of the binding sites must be different in the two receptors.^9^ While DC‐SIGN exists as a single form with seven full repeat units and one partial repeat unit, polymorphisms in the gene for DC‐SIGNR result in forms that differ in the number of neck repeat units^9^ and this variation is also correlated with differences in susceptibility to HIV infection.^10^

To facilitate analysis of the oligomer formation by DC‐SIGN and DC‐SIGNR, a strategy for purification of the neck domains has been developed, based on addition of the sequence Gly‐His‐His at the C‐terminus of the neck domain at the point where the CRD would normally be attached [Fig. 1(B)]. When the neck domain oligomerizes, the resulting cluster of histidine residues allows purification of the expressed polypeptides on immobilized nickel resin. Using this approach, the neck domain of each receptor has been shown to function as an oligomerization domain that can form tetramers in the absence of the flanking membrane anchor and CRD.^11^ The structure of the repeat units in the neck domain of DC‐SIGNR has been deduced from an unusual crystal form in which the unit cell comprises a single repeat unit in the context of a fragment containing seven repeat units.^5^ Each repeat unit consists of a short N‐terminal nonhelical region that includes a proline residue, followed by a coiled‐coil region. A heptad pattern of leucine, isoleucine and valine residues in the helical region mediates packing of the helices in a 4‐stranded coiled‐coil in the neck domain tetramer.

Although similar in organization, the neck domains of DC‐SIGN and DC‐SIGNR differ in important respects. For instance, the neck domain of DC‐SIGNR is much more stable to thermal denaturation than the neck domain of DC‐SIGN.^11^ These differences presumably reflect the effects of differences in the sequences in the repeat units in the two receptors. However, the way that individual differences in the neck domain repeat units affect the structure and stability of the neck domain has not been defined. Force measurements of the extracellular portions of both DC‐SIGN and DC‐SIGNR are consistent with the presence of the 37‐Å‐long 23‐amino acid repeat units in an extended structure.^12^, ^13^ However, no detailed structural information for the neck domain of DC‐SIGN is available.

The goal of the present studies was to analyze the properties of engineered versions of the neck domains from DC‐SIGN and DC‐SIGNR in order to understand the effects of sequence variations in the neck domains, both between the two proteins and between the different polymorphic forms of DC‐SIGNR. The analysis suggests that differences in key residues can result in differences in the assembly and organization of the extracellular portions of the receptors.

## Results

### Oligomerization of DC‐SIGN and DC‐SIGNR through the neck domains

Two different crystal structures of the C‐terminal portion of DC‐SIGNR suggest that there are only limited interactions between the CRDs in the oligomer.^14^ Because similar crystals of DC‐SIGN have not been obtained, an alternative approach was used to confirm that the presence of the CRDs has little influence on formation of tetramers in DC‐SIGN. In these experiments, the ability of the isolated neck domain to interact with the full extracellular portion of the receptor was investigated. If there were significant interactions between the CRDs that stabilize the tetramers, it would be expected that these additional interactions would favor formation of homo‐oligomers of the fragment representing the full extracellular portion of the receptor rather than mixed oligomers containing the neck domain fragments and the full extracellular fragment.

**Figure 2:**
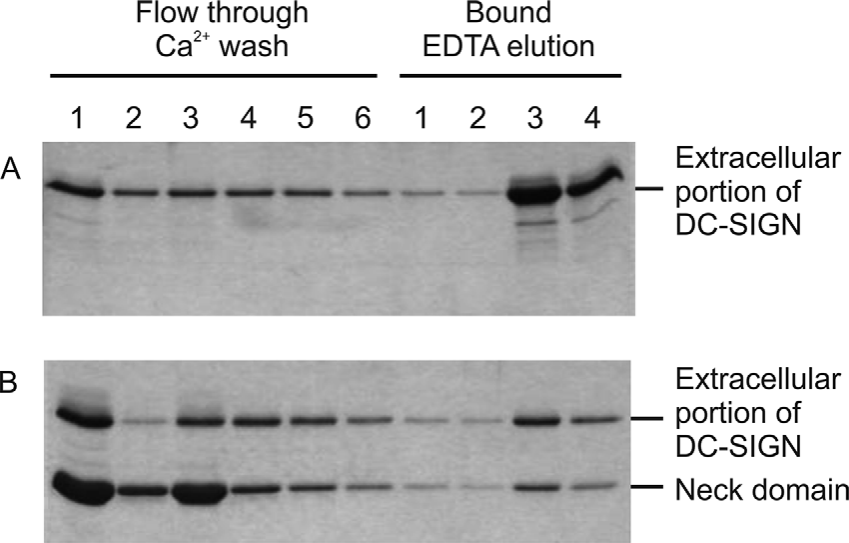
SDS‐polyacrylamide gel electrophoresis of neck domain co‐purification with the extracellular portion of DC‐SIGN on mannose‐Sepharose. The extracellular fragment of DC‐SIGN alone or the extracellular fragment and the neck domain fragment mixed in a ratio of ∼4:1 by weight were dissolved in 8 M Urea containing 50 mM Tris, pH 6.8, and dialyzed extensively against Ca^2+^‐containing loading buffer. The renatured samples were fractionated on a 1‐mL column of mannose Sepharose that was washed with four 1‐mL aliquots of Ca^2+^‐containing loading buffer and eluted with four 0.5‐mL aliquots of EDTA‐containing elution buffer. Aliquots were run on a 17.5% SDS‐polyacrylamide gel, which was stained with Coomassie blue.

When the purified neck domain and the extracellular fragment of DC‐SIGN were mixed, denatured and allowed to renature, fractionation of the resulting renatured protein on a mannose‐Sepharose affinity column results in both fragments being retained on the column and eluted with EDTA. Because the neck domain cannot bind to mannose‐Sepharose on its own, the results represent purification of mixed oligomers that contain both the neck domain and the full extracellular fragment (Fig. 2). The fact that the two different polypeptides associate in an apparently random fashion is consistent with the interpretation that the formation of tetramers of DC‐SIGN is independent of interactions between the CRDs and is dictated by the sequences of the neck domain.

Some information about the effects of variations in the sequences of the neck repeat units can be derived from earlier studies. The N‐terminal portions of the extracellular portions of DC‐SIGN and DC‐SIGNR consist of a short region not related to the 23‐amino acid repeat units, followed by a half repeat unit that is significantly divergent from the other repeat units. In previous studies, truncation of the neck domain of DC‐SIGNR to remove the non‐repeat region at the N‐terminus plus the partial repeat unit at this end resulted in no change in the stability of the neck domain, since the observed denaturation temperature measured by differential scanning calorimetry remained at 82°C.^5^, ^11^ Similar truncation of the neck domain of DC‐SIGN results in a 7‐repeat fragment that remains a tetramer at room temperature and the denaturation temperature of 56°C measured by calorimetry is very close to the value of 54°C for the full length neck domain previously reported (data not shown).^11^ Thus, for both DC‐SIGN and DC‐SIGNR, the stability of the tetramers is largely a function of the 7 full repeat units at the C‐terminus of the neck domain.

### A stable prototype neck domain repeat unit

The most divergent sequence is the C‐terminal repeat unit of DC‐SIGNR, adjacent to the CRD [Fig. 1(C,D)]. The crystal structure of the neck domain did not reveal a contribution of the variant residues to the electron density of the average repeat unit, suggesting that the C‐terminal repeat unit is not ordered like the other repeat units in the crystal.^5^ In the structure of the CRD of DC‐SIGNR with a truncated portion of the neck domain, the four copies of the C‐terminal repeat unit were found to be splayed apart.^14^ A key feature of this C‐terminal repeat unit is a phenylalanine residue at position 20. In all other copies of the repeat unit, this position is occupied by a valine residue, which is one of the residues that forms part of the heptad motif of hydrophobic residues characteristic of coiled‐coil structures. The valine side chain is able to pack into the coiled coil, while the phenylalanine side chain is too large to be accommodated. Thus, the variation in this final repeat unit is associated with the special functions of this part of the neck domain in positioning the CRDs at a broad spacing.

Aside from this unusual C‐terminal repeat unit, a key point of variation between DC‐SIGN and DC‐SIGNR is at position 6 of the repeat units, which forms the first residue of the heptad pattern in the primary structure and the beginning of the α helix in the crystal structure. In all of the repeat units visible in the crystal structure of the DC‐SIGNR neck domain, corresponding to repeat units 2 through 7, this position is occupied by a leucine residue, which packs into the hydrophobic core of the coiled coil [Fig. 1(E)]. However, this leucine residue is present only in repeat units 2 through 4 of DC‐SIGN.

**Figure 3:**
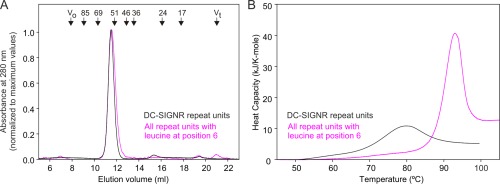
Stability of the neck domain consisting of seven repeat units, with leucine at position 6 of each repeat unit. (A) Gel filtration analysis of the engineered neck domain containing leucine residues at position 6 and arginine residues at position 15 in each repeat unit, compared to the previously characterized repeat region from the neck domain of DC‐SIGNR. (B) Differential scanning calorimetry of the DC‐SIGNR neck domain shows a denaturation temperature of 82°C,^5^ compared with a denaturation temperature of 94°C when leucine and arginine are present in each repeat unit. Protein concentrations were 230 and 94 μM for the natural polypeptide and the engineered protein, respectively.

To assess the effect of the residue at position 6 on the structure and stability of the neck domain, an engineered neck domain consisting of seven repeat units, each containing a leucine residue at position 6, was prepared. Gel filtration revealed that this version of the neck domain forms a stable tetramer at room temperature since it elutes at the same position as a natural fragment of the neck domain of DC‐SIGNR containing seven repeat units, which has been characterized as a tetramer [Fig. 3(A)].^5^ Analysis by differential scanning calorimetry showed that the uniform neck domain is actually more stable than the equivalent portion of the natural DC‐SIGNR neck domain, with a thermal denaturation temperature of 94°C compared to 82°C for the natural neck domain [Fig. 3(B)]. One reason for the lower stability of the natural neck domain would be the presence of the potentially destabilizing C‐terminal repeat unit. In addition, the uniform neck domain contains an arginine residue at each position 15, while this residue is glutamine or glutamic acid in several of the repeat units in the natural neck domain. In the crystal structure of the neck domain, the arginine residue is on the outer surface of the bundle of helices and forms a salt bridge with a conserved glutamic acid side chain at position 12 in the same polypeptide [Fig. 1(E)]. Such an i,i + 3 salt bridge hydrogen bond would contribute to stability of the repeat units.^15^, ^16^ Together, these results define a prototype sequence of a neck repeat unit with very high stability, in which leucine is present at position 6, packed on the inside of the coiled coil and arginine is present at position 15 making a salt bridge on the outside of bundle of helices.

### Effect of number of repeat units on oligomer formation

In addition to differences in stability reflecting minor variations in the sequences of the repeat units in the neck domains of DC‐SIGN and DC‐SIGNR, differences in the properties of the neck domains could arise from differences in the number of repeat units in polymorphic forms of DC‐SIGNR. To compare these factors, multiple versions of the neck domain were created with 3 to 7.5 copies of the 23‐amino acid repeat unit containing leucine at position 6 and arginine at position 15.

**Figure 4:**
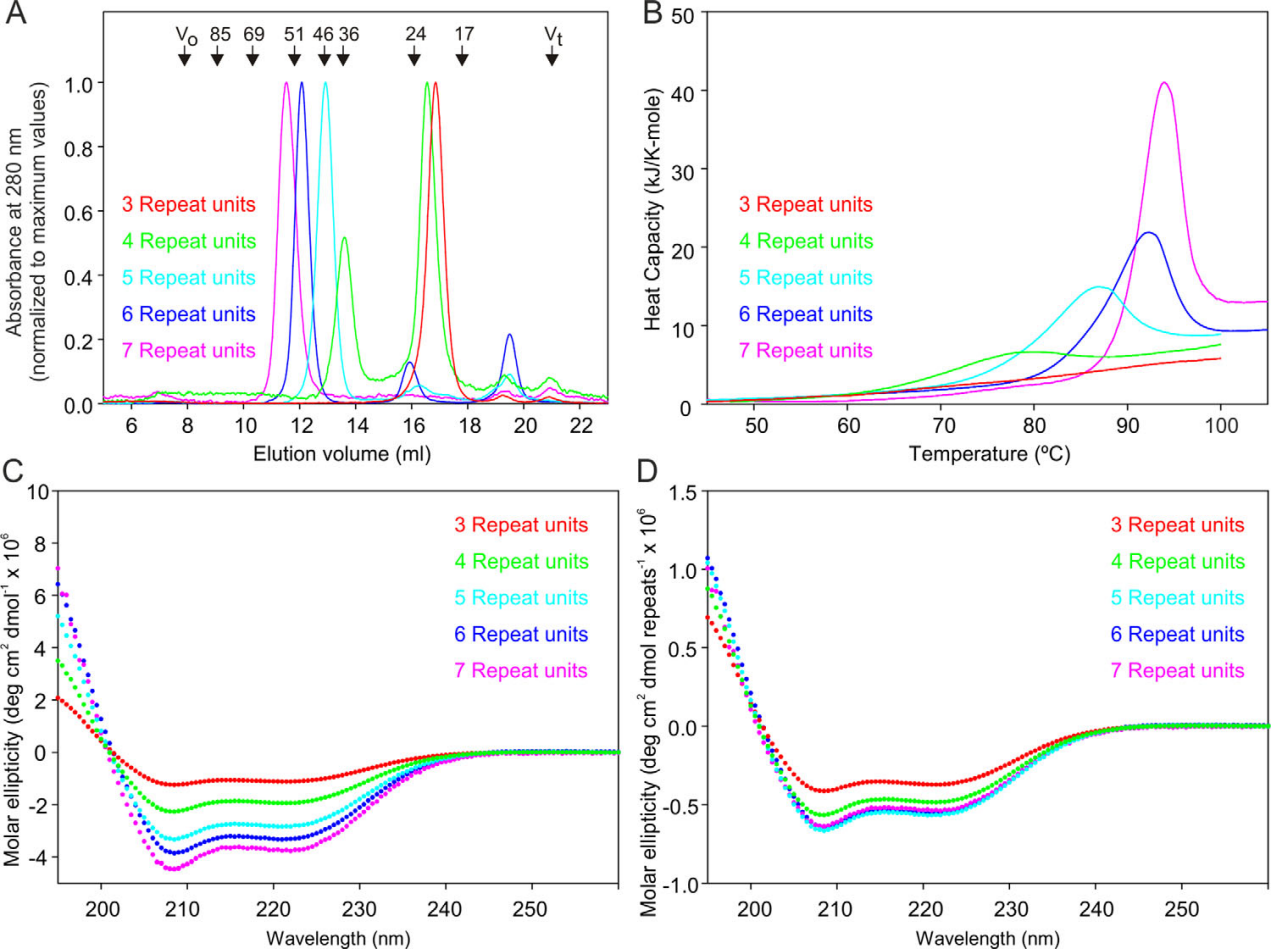
Analysis of the stability and conformation of uniform neck domains containing different numbers of repeat units with leucine at position 6. (A) Gel filtration analysis showing decreasing molecular weight of the tetramer for the shorter forms and dissociation of the two smallest versions. (B) Differential scanning calorimetry reveals reduced denaturation temperatures for the shorter forms of the neck. Protein concentrations were 210, 210, 220, 189, and 94 μM for the polypeptides with 3, 4, 5, 6, and 7 repeat units, respectively. (C) Circular dichroism spectra expressed per mole of polypeptide. Protein concentrations were 21, 16, 6.2, 6.7, and 10 μM for the polypeptides with 3, 4, 5, 6, and 7 repeat units, respectively. (D) Circular dichroism spectra normalized to number of repeat units to demonstrate that all spectra have the same shape and only the shortest forms differ in the intensity of the signals.

Changes in the stability of the neck domain oligomers were demonstrated by gel filtration analysis, which reveals a single peak of tetramers for the longer constructs, but the appearance of increasing amounts of monomer for shorter fragments containing 5 and 4 repeat units, while a fragment containing only three repeat units appears entirely as monomer [Fig. 4(A)]. Similar trends in stability were also observed by differential scanning calorimetry, in which a transition occurs at lower temperatures for the shorter constructs and is entirely absent for the version with three repeat units [Fig. 4(B)]. These experiments are performed at high concentrations that would favor tetramer formation, which makes it possible to observe unfolding of the tetramer in all cases except for the fragment with three repeat units. This shortest version thus shows no evidence of tetramer formation in either the gel filtration or calorimetry experiments.

The CD spectra of most of the constructs share the largely helical conformation of the natural neck domains of DC‐SIGN and DC‐SIGNR [Fig. 4(C)].^11^ Normalization of the values to the number of repeat units reveals that the signal per repeat unit for the longer fragments is constant [Fig. 4(D)], indicating that the repeat units have the same conformation as seen in the crystal structure. However, when the number of repeat units drops below 4, there is a change in the ratio of the signals at 208 and 222 nm. Values of this ratio higher than 1 are associated with small differences in the helical pitch resulting from packing in coiled‐coil structures.^17^ The expected changes are best characterized for parallel 2‐ and 3‐stranded coiled‐coils, but formation of the parallel 4‐helical bundle would be expected to have a similar effect. The slight decrease in the intensity of the CD signal per repeat unit for the neck domain with four repeat units is consistent with the gel filtration results showing that coiled‐coil tetramer is in equilibrium with a monomeric structure at the concentration used for gel filtration and CD analysis. The higher molar concentrations of the smaller fragments required to obtain good quality spectra would favor tetramer formation, so the results probably slightly under‐estimate the effect of partial dissociation for the fragment with 4 repeat units. In contrast, the fragment with only three repeat units shows a more significant loss of helical structure and a reduced ratio of the signals at 208 and 222 nm, which is consistent with the conclusion that this fragment does not form stable tetramers containing coiled coils. Taken together, these results indicate that a fully stable coiled‐coil structure requires the presence of at least five repeat units.

### Effects of variation in the sequences of repeat units on stability and conformation

A leucine residue is present at position 6 only in repeat units 2 through 4 of DC‐SIGN, with position 6 occupied by a glutamine residue in most of the remaining repeat units, although a methionine residue is present in repeat unit 5. To assess the effect of this variation at position 6 on the structure and stability of the neck domain, further engineered versions of the domain containing 7 repeat units with either glutamine or methionine at position 6 in each repeat unit were prepared.

**Figure 5:**
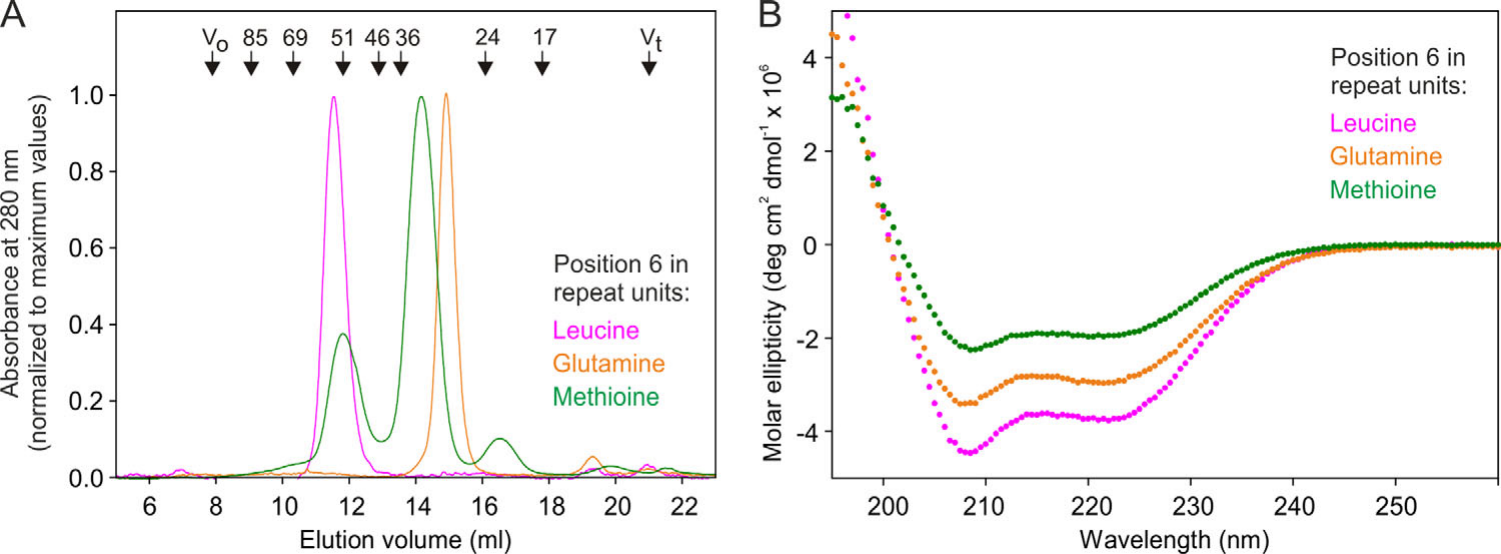
(A) Gel filtration analysis of proteins containing seven repeat units, with leucine, glutamine or methionine at position 6 of each repeat unit. (B) Circular dichroism analysis of the proteins consisting of seven identical repeat units in which each repeat unit contains leucine, glutamine or methionine at position 6. Protein concentrations were 10, 13, and 20 μM for the polypeptides with repeat units containing leucine, glutamine and methionine at position 6, respectively.

In contrast to the homogeneous neck domain containing leucine at position 6 of each repeat unit, otherwise identical neck domain in which glutamine is present at this position in each repeat unit fail to form stable oligomers. At room temperature, the all‐glutamine version of the neck domain elutes as a monomer on gel filtration [Fig. 5(A)]. This neck domain does not yield a clear denaturation transition by differential scanning calorimetry, which is consistent with the absence of stable oligomers (data not shown). Circular dichroism analysis revealed the presence of α helical structure, but the molar ellipticity is reduced compared to the neck domain containing leucine residues at position 6 of the repeat units [Fig. 5(B)]. The all‐glutamine version of the neck domain shows a ratio of signals at 208 compared to 222 nm comparable to that of the all‐leucine neck domain. These results suggest that the all‐glutamine version transiently forms coiled coils under the static conditions of the circular dichroism experiment, but that these oligomers are not stable enough to be observed in the gel filtration experiment.

An analog of the neck domain containing methionine residues at position 6 of each repeat unit also did not show a defined denaturation peak by calorimetry (data not shown) and has even more diminished helical content [Fig. 5(B)]. In this case, the ratio of molar ellipticity at 208 nm compared to 222 nm is reduced, suggesting that helix formation is not accompanied by formation of coiled coils like those seen in the other forms of the neck domain. The oligomeric state assessed by gel filtration is also poorly defined. It appears as multiple broad peaks that do not elute with either monomers or tetramers [Fig. 5(A)]. These results suggest that there may be at least transient formation of intermediate species in which the normal tetrameric coiled‐coil interactions are absent.

The combined results of the gel filtration, calorimetry and circular dichroism studies suggest that the presence of glutamine at position 6 of the neck domain repeat unit is compatible with formation of the tetrameric coiled coil, so that a repeat unit containing this sequence can have the same conformation as a repeat unit with leucine at position 6, although the glutamine form is less stable. In contrast, a methionine residue at this position disrupts the conformation.

A final unusual feature of the DC‐SIGN neck domain is a tryptophan residue at position 15 in the fourth repeat unit. This substitution has little effect on the behavior of the neck domain, as shown by the fact that substitution of the first portion of the neck domain from DC‐SIGNR, including an arginine residue at this position, for the corresponding portion of DC‐SIGN results in removal of the tryptophan residue with no detectable effect on the stability of the neck domain.^11^

### DC‐SIGN and DC‐SIGN variation between species

**Figure 6:**
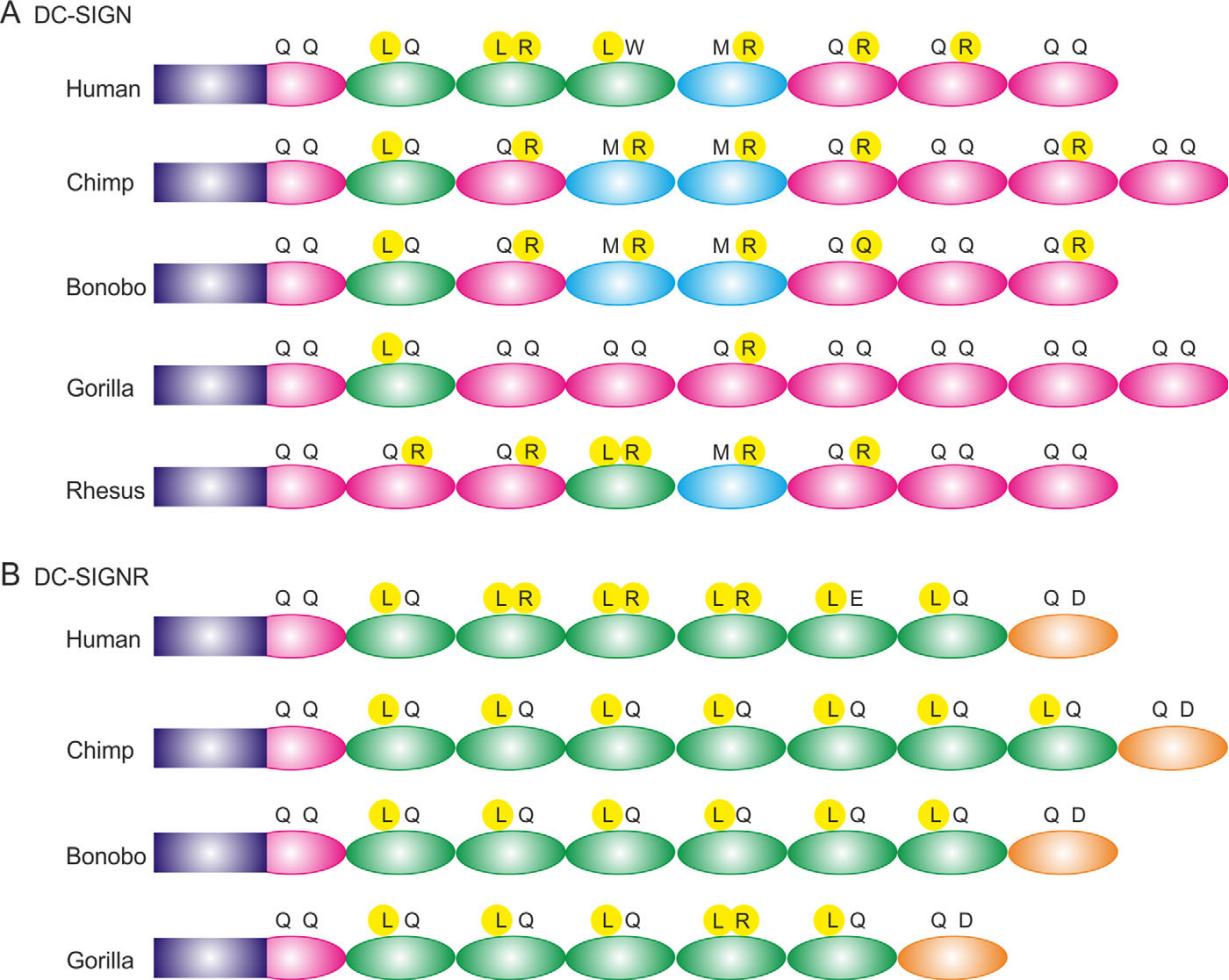
Comparison of the sequences of neck domains from DC‐SIGN homologs in various primates. Residues at positions 6 and 15 of the repeat units are indicated in single‐letter amino acid code, with those that stabilize the tetrameric coiled‐coil, leucine at position 6 and arginine at position 15, highlighted in yellow. Repeat units that contain leucine at position 6 are colored green, repeat units that contain glutamine at this position are colored pink and repeat units that contain methionine are colored cyan. The unusual repeat units at the C‐terminus of the neck domain of DC‐SIGNR are colored orange. Non‐repeat regions at the N‐terminus are indicated by purple rectangles. (A) DC‐SIGN neck domain organization. (B) DC‐SIGNR neck domain organization.

DC‐SIGN and its homologs are subject to rapid genetic change.^18^ The 23‐amino acid repeat units of the neck domain are present only in primates. A very divergent set of eight homologous genes is found in mice.^19^ Based on the roles of some of the key amino acids in the neck domain described here, comparison of the DC‐SIGN and DC‐SIGNR genes in the primates provides some insights into the potential functional effects of these genetic variations (Fig. 6). Some of the distinct structural features conserved in the DC‐SIGNR genes are the presence of the stabilizing leucine residue at position 6 of all but the first and last repeat units and the presence of the unusual phenylalanine residue at position 6 in the repeat unit adjacent to the CRD, although there is variation in the number of repeat units. In contrast, the DC‐SIGN sequences show greater diversity, although the more stable repeat units, with the leucine at position 6 and arginine at position 15, are generally found nearer to the membrane, while repeat units at the C‐terminal end, towards the CRD, mostly contain glutamine at position 6. The destabilizing methionine residue is present at position 6 in only one or two repeat units in the middle of the neck domain. Conservation of these patterns, in spite of variation in the number of repeat units and the specific sequences, suggests that these features are selected because they confer different functional properties in the two receptors.

## Discussion

The previously described crystal structure of the neck domain of DC‐SIGNR is based on an average from the different types of repeat units present. Each of these repeat units has a leucine residue at position 6, although they vary at position 15. The results can be assembled into a model for the full extracellular portion of DC‐SIGNR.^5^ It has not been possible to obtain crystals of the forms of the neck domains with glutamine or methionine residues at position 6 of the repeat units, or for the natural neck domain of DC‐SIGN containing a mixture of residues at position 6 of different repeat units, so the alternative strategy described here was to use spectroscopy, calorimetry, and gel filtration to suggest ways that sequence variation alters the structure from that observed for the repeat units with leucine residues at position 6.

Based on this combination of information, it is concluded that repeat units with leucine residues at position 6 and arginine residues at position 15 form particularly stable tetrameric coiled coils. Changing the arginine residue at position 15 results in loss of the salt bridge with the conserved glutamic acid residue at position 12, which causes some destabilization, while introduction of alternative residues at position 6 is more destabilizing. The summary of the sequences of DC‐SIGN and DC‐SIGNR molecules shown in Figure 6 reveals that relatively few repeat units are stabilized by the presence of both a leucine residue at position 6 and an arginine residue at position 15, but a high proportion of the repeat units contain one or the other of these residues. Appropriate residues at either one of these two positions may be alternative ways of achieving stability of the coiled coil.

The loss of stability resulting from the presence of a glutamine residue at position 6 is somewhat surprising, since favorable packing and hydrogen bonding of glutamine residues has been observed in trimeric coiled coils^20^ and the glutamine side chain can be accommodated sterically in the interior of the tetrameric coiled coil. The circular dichroism results confirm that the presence of glutamine is compatible with the coiled coil structure, with the decreased signal reflecting the loss of stability so that the tetramer is dissociated more of the time. It may be significant that the glutamine residue at position 6 would be the first residue of the coiled coil, following the disruption caused by the preceding proline and charged residues. The loss of hydrophobic interactions may be of greater significance in this context, leading to destabilization. In contrast, the presence of a methionine residue at position leads to more significant disruption of the structure of the neck domain, which may be explained based on steric constraints resulting from the longer, linear side chain that would not readily pack inside the coiled coil. Loss of stability and the potential disruption to the helical structure resulting from the presence of a methionine residue in the middle repeat unit probably explains why the neck domain of DC‐SIGN is resistant to crystallization.

For DC‐SIGNR, a key finding to emerge from these studies is the observation that the repeat units are mostly stabilized by leucine residues at position 6 and arginine residues at position 15. The presence of a phenylalanine residue at position 20 in the repeat unit adjacent to the CRD appears to cause a flaring apart of the CRDs, but the remainder of the neck domain is a stable tetrameric coiled coil. This arrangement of very stable repeat units in the neck domain, which is conserved in all of the DC‐SIGNR proteins in the primates, may be necessary to ensure that the neck domain remains tetrameric even though the final repeat units are splayed apart by the presence of the phenylalanine residue. The studies reported here suggest that the presence of stabilizing residues at positions 6 and 15 of the repeat units allows for the formation of stable tetramers even for shorter versions of the neck domain present in some individuals, which result from common genetic polymorphisms in the human population.^21^, ^22^ However, the results also define a minimal length for the neck domain to be fully stable, even in the presence of stabilizing residues within the repeat units. The very stable structure of the coiled coil ensures that the spacing between the CRDs in DC‐SIGNR is relatively fixed, although the exact orientation of the CRDs would be potentially flexible because of the flaring apart caused by the unusual repeat unit adjacent to the CRD.

In contrast to DC‐SIGNR, only the repeat units at the membrane‐proximal end of the neck domain in DC‐SIGN are stabilized by leucine residues at position 6. The presence of potentially less stable repeat units in the half of the DC‐SIGN neck domain further from the membrane, and the presence of repeat units containing the potentially disruptive methionine residues at position 6 in a repeat unit in the middle of the neck domain, raises the possibility that the C‐terminal portion of the neck domain might dissociate some of the time, so that the extracellular domain would be able to accommodate different spacings of sugar residues, as observed in recent studies with mannose‐coated particles.^9^ High affinity binding of lattices of sugars by multivalent receptors requires both multivalency and appropriate spacing and orientation of sugar‐binding sites,^23^, ^24^ so this arrangement would potentially allow the cluster of CRDs in the tetramer of DC‐SIGN to bind clusters of ligands at a greater range of spacings.

Some differences in the interactions of DC‐SIGN and DC‐SIGNR with glycans on pathogens may result from differences in the local sugar‐binding characteristics of the CRDs. For example, only DC‐SIGN binds to parasite glycoproteins because this binding results from recognition of the Lewis^x^ epitope, which is bound by DC‐SIGN but not DC‐SIGNR.^3^, ^25^ However, it is more difficult to explain differential binding of pathogens that bear high mannose oligosaccharides, as in the case of West Nile virus, which binds better to DC‐SIGNR than to DC‐SIGN, based on the interaction of individual oligosaccharides with the binding sites in the CRDs.^7^ It seems more likely that such differences result from different capacities of the receptors to accommodate various spacings of glycan epitopes resulting from the distinct properties of the neck domains described here.

## Materials and Methods

### Protein expression and purification

Procedures for expression of the extracellular portions of human DC‐SIGN and DC‐SIGNR in *Escherichia coli* followed by purification, employing affinity chromatography on mannose‐Sepharose, were as previously described.^26^ Wild type and mutated versions of the neck domain bearing 2‐histidine tags were purified by chromatography on immobilized Ni^2+^ followed by anion exchange chromatography.^5^, ^11^

### Generation of mutated neck domain polypeptides

**Figure 7:**
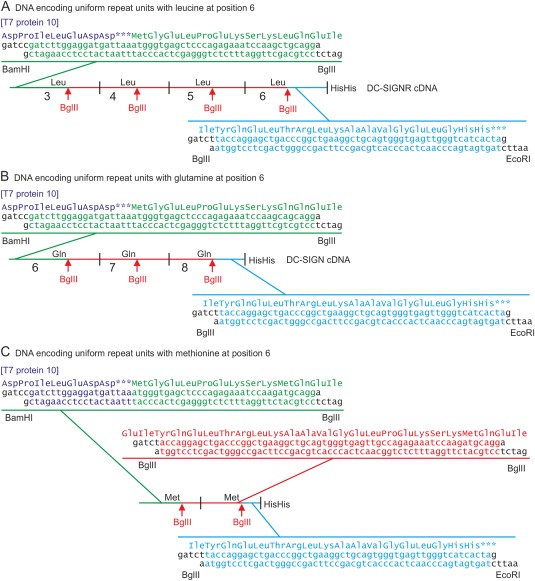
Strategies for generation of neck repeat units with uniform amino acid sequences. (A) Double‐stranded synthetic oligonucleotides used to replace the 5' and 3' ends of the portions of the DNA encoding repeat units 3 to 6 in DC‐SIGNR are indicated. (B) Oligonucleotides used to replace the ends of the cDNA encoding repeat units 6 to 8 of DC‐SIGN are shown. (C) A cDNA encoding two repeat units with methionine at position 6 was created by assembling three sets of synthetic oligonucleotides. In each case, the 5' BamH1 site allows insertion into the expression vector pT5T so that the neck polypeptide is translated when a ribosome restarts following termination of a truncated phage T7 gene 10 protein.^27^

Vectors encoding uniform neck domain repeat units with leucine at position 6 were created starting from the cDNA for the DC‐SIGNR neck.^5^ Synthetic oligonucleotides were used to replace the 5' sequence encoding the N‐terminus of repeat unit 3 and the 3' sequence encoding the C‐terminus of repeat unit 6, to create a DNA sequence encoding a block of 4 repeat units with identical amino acid sequences and a C‐terminal 2‐histidine tag [Fig. 7(A)]. Fragments encoding various numbers of repeat units from the 5' or 3' ends of this construct were created by partial digestion with BglII followed by digestion with the flanking enzymes BamHI or EcoRI, respectively. Pairs of 5' and 3' fragments were ligated to generate sequences encoding 3 to 7 repeat units. The resulting BamH1 to EcoR1 fragments were inserted into the T7 expression vector as for the wild type neck domains.^11^

A similar strategy was used to generate a DNA sequence encoding three repeat units with glutamine at position 6, by substituting synthetic oligonucleotides for the portions of the DC‐SIGN cDNA^11^ encoding the 5' end of repeat unit 6 and the 3' end of repeat unit 8 [Fig. 7(B)]. The BglII partial digestion strategy was used to expand the number of repeat units to 5 and then to 7.

For creation of neck domain polypeptides containing methionine residues at position 6, three sets of synthetic oligonucleotides were assembled to generate a DNA fragment encoding two repeat units [Fig. 7(C)]. Partial BglII digestion was repeated three times to expand the number of encoded repeat units first to 3, then to 5 and finally to 7 repeat units.

### Analytical procedures

Analytical affinity chromatography on mannose‐Sepharose^28^ was performed using loading buffer consisting of 0.5 M NaCl, 25 mM Tris‐Cl, pH 7.8, and 25 mM CaCl_2_ and elution buffer containing of 0.5 M NaCl, 25 mM Tris‐Cl, pH 7.8, and 2.5 mM EDTA. SDS‐polyacrylamide gel electrophoresis was performed in the buffer system of Laemmli.^29^ Gel filtration analysis employed a 1 × 30 cm^2^ Superdex S200 column eluted with 100 mM NaCl, 10 mM Tris‐Cl, pH 7.8, 2.5 mM EDTA. The positions of marker proteins are indicated by the Stokes radius: cytochrome c, 17 Å; bovine erythrocyte carbonic anhydrase, 23.9 Å; bovine serum albumin, 35.5 Å; yeast alcohol dehydrogenase, 45.5 Å; β‐amylase, 51 Å; E. coli β‐galactosidase, 69 Å; and thyroglobulin, 85 Å. Protein concentrations were determined by ninhydrin assay following alkaline hydrolysis.^30^

### Differential scanning calorimetry

Samples for calorimetry were dialyzed extensively against 125 mM NaCl, 25 HEPES, pH 7.8, 2.5 mM CaCl_2_, degassed under vacuum and introduced into the sample loop of a Nano‐III calorimeter from Calorimetry Sciences Corporation. Repeated equilibration scans from 5 to 20°C were used to remove any remaining dissolved gas. Scans were performed at 1°min^−1^. Protein concentrations used were based on those previously employed for the native neck polypeptides, since these give signals that can be quantified accurately.^11^ For experiments in which different proteins were compared, protein concentrations varied by less than three‐fold.

### Circular dichroism

Proteins were prepared in 125 mM NaCl, 25 mM Tris–Cl, pH 7.8, 5 mM CaCl_2_. Analysis was performed on 200 μL samples in a 1‐mm cuvette in a Chirascan spectropolarimeter from Applied Photophysics, with data collected for 1 sec at 0.5 nm intervals followed by averaging of 10 scans. Comparisons were made between proteins within a three‐fold range of concentrations. In test experiments over this concentration range, the ratio of tetramers to monomer observed on gel filtration changed by less than 1.5‐fold for the fragments that undergo some dissociation into monomers, such as the version containing four repeat units with leucine at position 6.
